# Association Between Freezing of Gait and Sleep Quality in People with Parkinson’s Disease

**DOI:** 10.3390/brainsci16050493

**Published:** 2026-04-30

**Authors:** Tracy Milane, Edoardo Bianchini, Lanfranco De Carolis, Antonio Suppa, Marco Salvetti, Clint Hansen, Massimo Marano, Domiziana Rinaldi, Nicolas Vuillerme

**Affiliations:** 1 Univ. Grenoble Alpes, CNRS, Grenoble INP, LIG Sangria, 38000 Grenoble, France; tracymilane@gmail.com (T.M.); nicolas.vuillerme@univ-grenoble-alpes.fr (N.V.); 2 Fondazione Policlinico Universitario Campus Bio-Medico, Via Alvaro del Portillo 200, 00128 Rome, Italy; 3 Department of Neuroscience, Mental Health and Sensory Organs (NESMOS), Sapienza University of Rome, 00189 Rome, Italymarco.salvetti@uniroma1.it (M.S.); 4 Department of Human Neurosciences, Sapienza University of Rome, 00185 Rome, Italy; antonio.suppa@uniroma1.it; 5 IRCCS Neuromed Institute, 86077 Pozzilli, Italy; 6 Department of Neurology, Kiel University, 24105 Kiel, Germany; c.hansen@neurologie.uni-kiel.de; 7 Unit of Neurology, Neurophysiology, Neurobiology and Psychiatry, Department of Medicine, Campus Bio-Medico University of Rome, 00128 Rome, Italy; 8 Institut Universitaire de France, 75005 Paris, France

**Keywords:** Parkinson’s disease, freezing of gait, sleep disorders, gait disorders, non-motor symptoms, sleep quality

## Abstract

**Highlights:**

**What are the main findings?**

- Freezing of gait (FOG) severity was positively associated with sleep disturbances in people with Parkinson’s disease experiencing FOG.
- Despite these associations, no significant differences in sleep quality or excessive daytime sleepiness were found between participants with and without FOG after propensity score matching.

**What are the implications of the main findings?**

- The co-occurrence of FOG and sleep disturbances may reflect, at least in part, shared neuroanatomical substrates, although this hypothesis requires confirmation in future mechanistic studies.
- FOG in Parkinson’s disease appears to be part of a broader pattern of motor complications and disease progression; these findings support the clinical relevance of considering both motor and non-motor symptoms in people with Parkinson’s disease with FOG.

**Abstract:**

**Background/Objectives**: Freezing of gait (FOG) and sleep disturbances are common in people with Parkinson’s disease (PwPD). A bidirectional association between them has been suggested, but quantitative evaluations are scarce. This study aimed to compare sleep disturbances in mild-to-moderate PwPD with (PD+FOG) and without FOG (PD−FOG), and to assess the association between FOG severity and sleep parameters. **Methods**: Data from 54 PwPD with disease stage <4 and no severe cognitive decline were included (27 PD+FOG and 27 propensity score-matched for age, sex, and disease duration PD−FOG). Demographics and clinical variables were collected. Clinical assessment included the new freezing of gait questionnaire (NFOG-Q), Parkinson’s Disease Sleep Scale (PDSS-2), Epworth Sleepiness Scale (ESS) and Movement Disorder Society Unified Parkinson’s Disease Rating Scale (MDS-UPDRS). Mann–Whitney U, Fisher’s exact and Spearman’s tests were used for group comparisons and correlations, respectively. **Results**: Significant differences were observed between PD+FOG and PD−FOG groups in MDS-UPDRS part II (*p* = 0.011) and part IV (*p* = 0.011), with higher scores in PD+FOG participants. No significant differences were found in PDSS-2 or ESS between the two groups. A significant moderate positive correlation was found between NFOG-Q score and PDSS-2 (ρ = 0.416; *p* = 0.044) in PD+FOG participants. **Conclusions**: FOG severity was positively associated with sleep disturbances within the PD+FOG group. However, no significant difference in sleep quality or excessive daytime sleepiness was found between PD+FOG and PD−FOG after propensity score matching. PD+FOG participants experienced more severe motor complications and greater impairment in daily activities compared to PD−FOG.

## 1. Introduction

Parkinson’s disease (PD) is a progressive and complex neurological disease and the second most common neurodegenerative disorder worldwide. PD is characterized by motor symptoms such as resting tremors, rigidity, and bradykinesia. As the disease progresses, axial motor symptoms including postural instability, gait problems and freezing of gait (FOG) may also develop [1].

FOG is a disabling symptom, defined as a brief, episodic absence or marked reduction in forward progression of the feet despite the intention to walk [2] and recently updated as “paroxysmal episodes wherein there is an inability to step effectively, despite attempting to do so” [3]. People with PD (PwPD) often describe it as if their feet suddenly become “glued” to the ground when they try to move forward [4]. It is estimated that FOG affects approximately 40% of PwPD (95% CI 35.3–44.5%), with prevalence varying widely depending on disease duration and severity, ranging from 22.4% in patients with disease duration <5 years to 70.8% in those with disease duration ≥10 years, and from 28.4% in mild stages to 68.4% in more advanced stages [5]. Typically, FOG episodes last a few seconds but can sometimes exceed 30 s [2]. These episodes are mainly triggered by certain provocative circumstances such as initiating gait, turning, navigating through narrow passages, or approaching a destination (e.g., a chair) [6]. Other triggering situations include performing dual-tasking, approaching doorways, or being in crowded spaces [2]. FOG can lead to falls in PwPD, severely restricting their independence, mobility and participation in social activities [4].

Besides motor symptoms, PD manifestations also include a wide set of non-motor symptoms (NMS), including urinary and gastrointestinal tract problems, cognitive impairment, autonomic dysfunction, sleep alterations, mood disorders, and hallucinations [1]. These could occur years before motor symptoms, and some NMS have been listed among the prodromal features of PD (i.e., rapid eye movement [REM] sleep behavior disorder [RBD], depression, olfactory loss) [1]. Previous studies reported an association between FOG and NMS, such as urinary symptoms, depression, and higher disease stage, as well as an association with NMS severity [7].

Among the NMS, sleep disorders are highly prevalent, with around 70% of PwPD reporting them [8]. These disturbances combine nocturnal and diurnal manifestations, which include insomnia, restless legs syndrome, RBD, parasomnias, sleep apnea and excessive daytime sleepiness (EDS) [9]. RBD can precede the onset of classic PD symptoms, while EDS typically comes in the later stages of the disease [10]. Indeed, sleep disorders not only negatively impact sleep quality but also affect daytime activities, further reducing participants’ quality of life and impairing both motor and cognitive functions [9]. Therefore, addressing and managing sleep disturbances could potentially help improve the well-being of PwPD.

Recently, there has been a growing interest in investigating the relationship between sleep disorders and FOG in PD. Indeed, identifying and understanding this relationship could result in more effective management strategies that might improve the quality of life of PwPD. Our recent systematic reviews [11,12] have suggested a probable bidirectional association between sleep disturbances and FOG in PD. On one hand, our findings revealed that PwPD with FOG tend to experience worse sleep quality, EDS, and more disruptive sleep disturbances compared to those without FOG [11]. On the other hand, PwPD with sleep disturbances had a higher prevalence of FOG compared to those without sleep disturbances [12]. Despite these findings, several key gaps remain in the literature. First, existing studies comparing sleep outcomes between PD+FOG and PD−FOG have relied on heterogeneous and often non-PD-specific assessment tools. Second, few studies have employed rigorous matching or adjustment strategies to control for key confounders, such as age, sex, and disease duration, that independently influence both FOG prevalence and sleep disturbances, making it difficult to isolate the specific contribution of FOG to sleep outcomes. Third, quantitative assessments of the association between FOG severity and sleep disturbance severity remain scarce [11,12]. Further research is hence still needed to better understand the underlying mechanisms and explore whether improving sleep quality could reduce FOG and vice versa.

To address these gaps, the present study had two main objectives: (1) to compare sleep disturbances in participants with (PD+FOG) and without FOG (PD−FOG) using a propensity score matching, and (2) to assess the association between FOG severity and sleep parameters in PwPD. Based on the findings of our systematic reviews [11,12], we hypothesized that participants with FOG would exhibit greater sleep disturbances compared to those without FOG, and that FOG severity would be positively associated with the severity of sleep disturbances.

## 2. Materials and Methods

This was a propensity score-matched analysis conducted on a cross-sectional dataset of 154 mild-to-moderate PwPD collected to validate wearable-based measures and investigate the interaction between mobility, motor and non-motor symptoms in PwPD. This cross-sectional study was conducted and reported in accordance with the STROBE guidelines. This analysis was aimed at comparing sleep disturbances in PD+FOG and PD−FOG and to assess the association between FOG severity and sleep parameters, as well as other clinical variables in mild-to-moderate PwPD.

### 2.1. Ethics

The study was performed in accordance with the ethical standards as laid down in the 1964 Declaration of Helsinki and its later amendments. Approval was granted by the local Ethical Committee of Sapienza University of Rome, Italy (Ref. 0372/2022 of 10/05/2022). Data collection and processing followed the current European regulation for data protection. All participants provided written informed consent before the beginning of measurements.

### 2.2. Study Participants

Participants were consecutively screened and recruited during scheduled visits at the Movement Disorder Outpatient Service of the Sant’Andrea University Hospital (Rome, Italy) between March 2023 and March 2026. Inclusion criteria were: (i) diagnosis of idiopathic PD according to MDS criteria [13]; (ii) age 18 years or older; (iii) disease stage <4 according to the modified Hoehn and Yahr scale (mHY) (“severe disability; still able to walk or stand unassisted”) [14], in order to ensure independent or minimally assisted ambulation, safety during assessments, and sufficient functional capacity to complete gait-related experimental procedures; (iv) ability to perform the experimental procedure. Exclusion criteria were: (i) severe cognitive impairment as defined by a Montreal Cognitive Assessment (MoCA) score < 18; (ii) orthopedic, rheumatologic, or systemic conditions affecting mobility as judged by the assessor.

### 2.3. Clinical Assessments

Participants were evaluated during scheduled visits when demographics (age, sex) and clinical measures, including disease duration, disease stage according to the mHY scale, and Levodopa Equivalent Daily Dose (LEDD), were collected. Participants were then evaluated with the following rating scales. The Movement Disorder Society–Unified Parkinson’s Disease Rating Scale (MDS-UPDRS) [15] parts I-IV was used to assess the impact of motor and non-motor symptoms on daily life, motor symptoms severity and motor complications, respectively, and the total score of MDS-UPDRS was calculated. The presence of motor fluctuations was defined as an MDS-UPDRS part IV score ≥ 1. The Parkinson’s Disease Sleep Scale, second version (PDSS-2) [16] and the Epworth Sleepiness Scale (ESS) [17] were used to assess sleep problems and EDS, respectively. A cut-off score ≥ 15 at PDSS-2 [18] and ≥10 at ESS [17] were used to define clinically significant sleep problems and EDS, respectively. The new freezing of gait questionnaire (NFOG-Q) [19] was used to assess the severity and presence of FOG as defined by a score ≥1 at item 1.

### 2.4. Statistical Analyses

Statistical analyses were performed using JASP v0.95.2 (JASP Team, University of Amsterdam, Amsterdam, The Netherlands), R v4.5.2 (R Foundation for Statistical Computing, Vienna, Austria) and RStudio 2026.01.1 Build 403 for Windows (Posit PBC, Boston, MA, USA). Descriptive statistics were calculated for the examined variables. Normality of distributions was assessed by histogram and residual plots inspection. From the total PwPD dataset, a nearest-neighbor propensity score matching for age, sex, and disease duration was performed using a custom script and the MatchIt package in R, with a 1:1 ratio between PD+FOG and PD−FOG participants, a logistic regression-estimated propensity score (logit link), and a caliper of 0.2 standard deviations of the logit of the propensity score. Balance across matching variables was assessed using the standardized mean difference (SMD), with SMD ≤ 0.10 considered indicative of adequate balance. The Mann–Whitney U test was used to assess differences in the examined variables between PD+FOG and PD−FOG participants after matching. Effect sizes were quantified using the rank-biserial correlation coefficient (r), with 95% confidence intervals estimated via stratified non-parametric bootstrap resampling within each group (2000 iterations, percentile method). Fisher’s exact test was used to compare the prevalence of wearing-off, sleep disturbances, and EDS between PD+FOG and PD−FOG. Within the overall PD+FOG group (N = 30), Spearman’s rank correlation was used to explore the relationship between FOG severity (NFOG-Q) and sleep outcomes (PDSS-2, ESS). The cut-off values used in the interpretations of correlation coefficients were: <0.1: negligible; 0.1–0.2: very weak; 0.2–0.4: weak; 0.4–0.7: moderate; 0.7–0.9: strong; >0.9: very strong [20]. The baseline significance threshold was set at α < 0.05, and Benjamini–Hochberg correction with a false discovery rate of 0.05 was applied to control for multiple testing. All data were reported as mean ± standard deviation or median (Q1–Q3) for numerical data and N (%) for categorical variables.

## 3. Results

Data from a total of 154 PwPD were included, of whom 30 (19.5%) presented with FOG (PD+FOG) and 124 (80.5%) did not (PD−FOG). Following propensity score matching for age, sex, and disease duration (nearest-neighbor 1:1, caliper = 0.2 SD of the logit of the propensity score), 27 PD+FOG participants were successfully matched to 27 PD−FOG controls, yielding a final matched sample of 54 participants. Three PD+FOG participants (10.0%) could not be matched within the specified caliper and were excluded from subsequent comparison analyses. Prior to matching, the PD+FOG and PD−FOG groups differed substantially in disease duration (SMD = 0.915), while age and sex were broadly comparable (SMD = 0.041 and 0.189, respectively). Following propensity score matching, SMD values were reduced across all three covariates (age: SMD = 0.140; sex: SMD = 0.081; disease duration: SMD = 0.047). Demographic and clinical characteristics of the enrolled population and of PD+FOG and PD−FOG participants are reported in Table 1.

### 3.1. Comparison Between PD+FOG and PD−FOG

**Figure 1:**
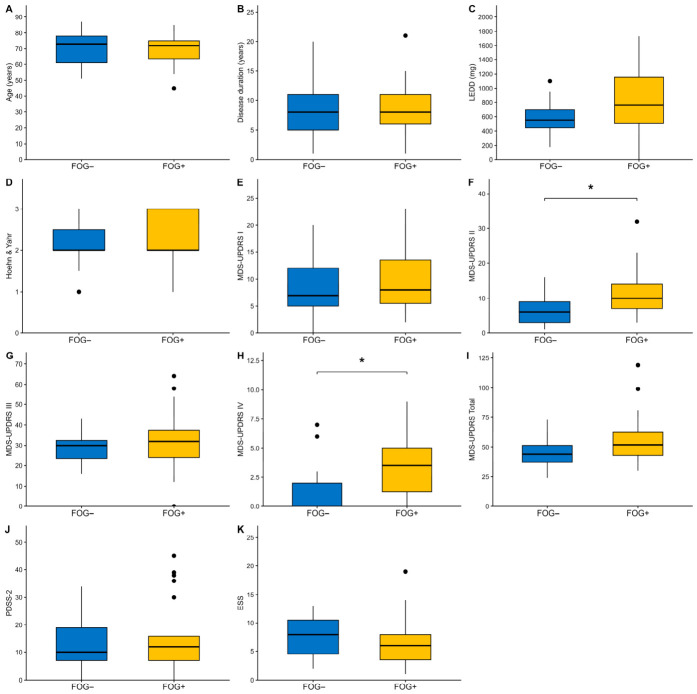
Boxplots showing differences in clinical score between PwPD with (FOG+) and without FOG (FOG−). (**A**) Age; (**B**) Disease duration; (**C**) LEDD; (**D**) mHY; (**E**) MDS-UPDRS Part I score; (**F**) MDS-UPDRS Part II score; (**G**) MDS-UPDRS Part III score; (**H**) MDS-UPDRS Part IV score; (**I**) MDS-UPDRS Total score; (**J**) PDSS-2 score; (**K**) ESS score; all compared between PwPD with (FOG+) and without FOG (FOG−). The thick line in the boxes indicates the median; lower and upper box limits indicate the first (Q1) and third quartile (Q3), respectively; vertical lines indicate lower and upper outliers’ boundaries calculated as Q1 − (1.5*IQR) and Q3 + (1.5*IQR), respectively. Significance is marked by a bracket and an asterisk (* *p* < 0.05, Benjamini–Hochberg corrected). ESS: Epworth Sleepiness Scale; FOG: freezing of gait; LEDD: levodopa equivalent daily dose; MDS-UPDRS: Movement Disorder Society Unified Parkinson’s Disease Rating Scale; mHY: modified Hoehn and Yahr scale; PDSS-2: Parkinson’s Disease Sleep Scale; PwPD: people with Parkinson’s disease.

The Mann–Whitney U test on the matched sample showed no significant difference between the two groups in PDSS-2 (*p* = 0.676) or ESS score (*p* = 0.288) after Benjamini–Hochberg correction. Significant differences were found in MDS-UPDRS part II (*p* = 0.011) and MDS-UPDRS part IV (*p* = 0.011), with higher scores in PD+FOG participants. LEDD showed a trend toward significance that did not survive correction for multiple comparisons (*p* = 0.091). No other significant differences were observed between the two groups, including MDS-UPDRS part I, part III, total score, Hoehn and Yahr stage, age, and disease duration (Table 1, Figure 1).

Regarding categorical variables, Fisher’s exact test showed a higher prevalence of fluctuations in PD+FOG compared to PD−FOG (74.1% vs. 40.7%, OR = 4.04, 95% CI 1.15–15.61), although this did not reach significance after Benjamini–Hochberg correction (*p* = 0.081). No significant differences were found between the two groups in the prevalence of sleep disturbances (33.3% vs. 37.0%, *p* = 1.000) or EDS (18.5% vs. 33.3%, *p* = 0.528) (Table 1).

### 3.2. Correlations Between FOG Severity and Sleep

**Figure 2:**
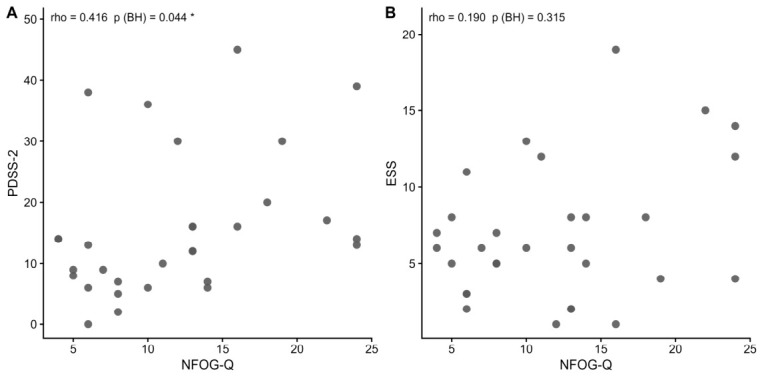
Scatterplot showing correlation between NFOG-Q and the other sleep variables in PD+FOG participants. Panel (**A**): correlation between NFOG-Q and PDSS-2. Panel (**B**): correlation between NFOG-Q and ESS. Spearman correlation coefficient and *p*-value after Benjamini–Hochberg correction are reported. BH: Benjamini–Hochberg; ESS: Epworth sleepiness scale; NFOG-Q: New freezing of gait questionnaire; PDSS-2: Parkinson’s disease sleep scale. Significance is marked by a bracket and an asterisk (* *p* < 0.05, Benjamini–Hochberg corrected).

Concerning correlations between FOG severity and sleep clinical variables in PD+FOG participants, Spearman’s test showed a significant moderate positive correlation between NFOG-Q score and PDSS-2 (ρ = 0.416; *p* = 0.044) (Table 2, Figure 2), indicating that greater FOG severity was associated with worse subjective sleep quality. No significant correlation was found between NFOG-Q score and ESS (ρ = 0.190; *p* = 0.315).

## 4. Discussion

This study aimed to compare sleep disturbances in participants with and without FOG and evaluate the association between FOG severity, sleep parameters, and clinical variables in mild-to-moderate PwPD. Data from 54 PwPD were analyzed, including 27 with FOG and 27 propensity-matched controls without FOG. The main finding of this study is that NFOG-Q scores are positively correlated with the PDSS-2 scores. Additionally, significant differences were observed between PD+FOG and PD−FOG groups in MDS-UPDRS part II and part IV.

### 4.1. Sleep Disturbances in PD+FOG and PD−FOG and Association with FOG

Our findings revealed a moderate positive correlation between NFOG-Q and PDSS-2 scores, indicating an association between self-reported FOG severity and sleep disturbances in PwPD reporting FOG. This study adds evidence on sleep disturbances in PD participants with and without FOG by employing the PDSS-2, a disease-specific tool, to assess sleep disturbances in this population. This correlation shows that as FOG severity increases, sleep disturbances become more pronounced. As it stands, our result corroborates those of De Almeida and colleagues [21], who observed a significant correlation between NFOG-Q scores and Pittsburgh Sleep Quality Index (PSQI) scores in 79 PwPD with (N = 40) and without (N = 39) FOG. These authors also found that the PSQI scores were the only predictor of the variance of the NFOGQ scores (R^2^ = 0.46, *p* < 0.0001) in the multiple regression analysis. Our results confirm this previous result and expand it to sleep disturbances assessed using a disease-specific tool such as PDSS-2. This evidence highlights that sleep problems and FOG possibly share a common underlying neurodegenerative mechanism observed in association with their occurrence [22]. Indeed, the symptoms of PD are caused by dysfunctions in different brain structures, disrupting various control areas, and resulting in both motor and non-motor impairments [1]. Among others, abnormalities in the brainstem could be crucial in the interaction between sleep disturbances and FOG. In particular, the pedunculopontine nucleus (PPN), a part of the mesencephalic locomotor center, plays a crucial role in the neuroanatomical network associated with gait control, locomotion and REM sleep [23]. Furthermore, the PPN is involved in regulating sleep–wake cycles and may also be involved in integrating gait control and sleep functions [24,25]. Research supports this connection, suggesting that FOG may be related to impairments in the brainstem neuroanatomical networks, particularly the PPN [26]. For instance, Snijders and colleagues [27] demonstrated that PD+FOG exhibit more pronounced atrophy in PPN gray matter compared to PD−FOG. Similarly, a reduced volume of the fiber tracts was observed in the right PPN of the PD+FOG participants compared to PD−FOG and to healthy controls [28]. These deficits can disrupt both motor and non-motor functions, suggesting that dysfunction in these regions and their neuroanatomical connections can alter both sleep and movement, potentially leading to the occurrence of sleep disorders in PD+FOG participants [23]. Indeed, functional deficits linked to RBD and FOG in PD have been observed in various neural networks, including the corticothalamic hypothalamic network, connections between PPN and cerebellar motor areas, PPN and auxiliary motor areas, and PPN to the bilateral medial prefrontal cortex and to the anterior cingulate cortex [23]. It must be emphasized, however, that we did not directly assess brainstem or PPN structure or function in our cohort. Any neuroanatomical interpretation should therefore be regarded as speculative and inferred from the previous literature, and warrants confirmation through dedicated neuroimaging or neurophysiological investigations.

Our findings did not reveal any significant differences in PDSS-2 score between PD+FOG and PD−FOG participants. This contrasts with the results of a previous study from Lv and colleagues [29], who reported a significant difference in PDSS score between PD+FOG and PD−FOG participants. However, after adjusting for confounding factors (i.e., age, BMI, educational level, disease duration, LEDD of Amantadine and Levodopa, mHY), the difference in PDSS became non-significant, aligning with our findings. Furthermore, it must be noted that the authors used the older version of the PDSS scale, which incorporated a different scoring method than PDSS2. In contrast to our findings, previous evidence evaluating specific sleep disorders has shown that PD+FOG people exhibited more severe symptoms of RBD [30,31], increased daytime sleepiness [30,31,32,33], and decreased sleep quality [21,32,34] compared with PD−FOG participants. However, all these previous works used different instruments to evaluate sleep problems, such as non-PD specific scales (i.e., PSQI [34], RBD screening questionnaire [30,31,34]) and the first version of PDSS, which encompasses different items than PDSS2 and a different scoring system [21,32]. This, together with the propensity-matching method used, could explain the differences between the results of our study and those from other previous studies.

Previous studies, also using the older PDSS version, reported that sleep disturbances were more prevalent in PD+FOG [31,32]. In our study, we did not find any significant difference in the prevalence of sleep disturbances between PD+FOG and PD−FOG participants, as indicated by a score ≥ 15 at PDSS-2 [18]. This discrepancy could be explained by the low number of people matching the cut-off score to define the presence of clinically relevant sleep problems in our study. Moreover, all the previous studies used item 1 of PDSS to define the presence of sleep disturbances; therefore, a different classification method was used.

The significant positive correlation between NFOG-Q and PDSS-2 scores within the PD+FOG group, in the absence of significant between-group differences in PDSS-2, may be interpreted as reflecting two complementary aspects of the FOG-sleep relationship. The between-group analysis addresses whether the presence of FOG as a binary phenotypic feature is associated with worse sleep quality. The within-group correlation, performed on the PD+FOG population, examines whether, among participants experiencing FOG, greater FOG severity covaries with worse sleep quality. The convergence of a non-significant group comparison and a significant severity-based association may suggest that, rather than being linked to the mere presence of FOG, sleep disturbances may be more closely related to FOG severity, potentially reflecting a shared involvement of common neuroanatomical substrates such as the brainstem and the PPN [23,24,25,26,27,28]. However, it should be acknowledged that this correlation is of moderate magnitude with borderline statistical significance and was based on a relatively small sample, increasing the risk of type I error. In addition, it is important to mention that the small sample size may have limited the ability to detect clinically meaningful differences. The null findings should therefore not be interpreted as solid evidence of the absence of effect, and our results should be regarded as exploratory and hypothesis-generating. Similarly, our interpretation should be taken cautiously due to its speculative nature and should be confirmed in larger longitudinal cohorts.

PwPD often suffer from EDS, which occurs following sleep disturbances at night, non-restorative night sleep, as well as due to disease-related factors and treatment [35]. It has been reported that the development of EDS is linked to the degeneration of the locus coeruleus and the ascending reticular activating system [30]. In addition, noradrenergic deficits due to cell loss in that area are possibly associated with falls, balance impairment, and FOG occurrence. In our analysis, we did not find any significant difference in ESS scores between PD+FOG and PD−FOG participants, as well as the prevalence of EDS. This result is consistent with three previous studies [36,37,38] in which no significant difference in ESS scores was observed between PD+FOG and PD−FOG in 20 [36], 62 [37], and 112 [38] PwPD, respectively. However, other studies [29,30,31,32,33] have reported that PD+FOG participants experienced more EDS compared to PD−FOG participants. One study found that EDS may serve as a potential prognostic indicator for the progression of FOG [39]. The difference with our results could be explained by the limited prevalence in our population of clinically relevant sleep disturbances (19/54) as well as of clinically relevant EDS, as indicated by an ESS score ≥10 (14/54).

### 4.2. Other Clinical Features in PD+FOG and PD−FOG

Our results showed that PD+FOG had significantly higher scores on the MDS-UPDRS II and IV compared with PD−FOG, suggesting they experience more severe motor complications and greater motor impairment in daily activities. Importantly, since the two groups were matched for disease duration, these differences cannot be attributed to longer disease exposure alone, suggesting that FOG may reflect a more complex disease phenotype, although residual confounding from other disease-related factors cannot be excluded. Our results are in line with previously published studies [29,31,33,40]. Xu and colleagues [31] recently reported that PD+FOG had a higher total MDS-UPDRS score, whereas Zhang and collaborators [33] and Lv et al. [29] reported that PD+FOG had higher scores on the MDS-UPDRS II and IV. Lichter and colleagues [40] found that compared to PD−FOG, PD+FOG had more motor complications, and that FOG occurrence was related to the severity of motor complications. These authors suggest that the relationship between FOG and motor complications might be partially explained by the increased motor dysfunction during “off” periods [40]. This is further supported by the trend toward a higher prevalence of motor fluctuations in PD+FOG compared to PD−FOG (74.1% vs. 40.7%), consistent with the notion that FOG episodes are more commonly observed during OFF states and may represent a manifestation of motor fluctuations. It should be noted that MDS-UPDRS part II includes a specific item on FOG, which may partially contribute to the higher scores observed in PD+FOG. Nevertheless, the overall higher MDS-UPDRS II score suggests that the functional impact of FOG extends beyond the freezing episodes themselves, affecting broader daily activities.

### 4.3. Limitations and Future Perspectives

We acknowledge that the current study has some limitations that should be considered. First, the small sample size limited statistical power and may have hampered the detection of subtle between-group differences, increasing the risk of type II errors and limiting generalizability. Studies with larger sample sizes are hence necessary to confirm and extend these findings. Second, the cross-sectional design of this study does not allow for establishing causal relationships between variables, and, in particular, does not allow for drawing conclusions about the directionality of the association between sleep disorders and FOG, nor about the causal role of neuroanatomical alterations, such as brainstem dysfunction, in driving either symptom. Longitudinal studies are needed to identify and observe changes in symptoms over time to better understand the relationship between sleep disturbances and FOG. Third, whereas we collected information on overall sleep disturbances and EDS, we did not specifically assess other sleep disturbances that are common in PD, such as RBD, insomnia, restless legs syndrome and disordered breathing [9]. Moreover, only a few participants have reported clinically bothersome sleep disorders [N = 19 (35%)] and EDS [N = 14 (26%)], which may have further reduced our ability to detect group differences. Therefore, although we consider our results relevant in the field, we could not draw a definitive conclusion on the association between specific sleep disturbances and FOG. Future studies are hence needed since each of these disorders may be associated differently with FOG and may have a different impact on PwPD. Fourth, the inclusion of PwPD with mHY < 4 and preserved cognition introduced a selection bias toward less advanced disease, reducing the generalizability of our findings to PwPD with more severe motor or cognitive involvement, in whom both sleep disturbances and FOG are typically more prevalent and severe [6,8,10,41]. Future studies, including participants with lower functional scores and worse cognitive conditions, may be needed. Fifth, sleep disturbances and FOG were evaluated using self-reported subjective scales (PDSS-2 and NFOG-Q), which carry important measurement limitations. Self-reported measures are inherently subject to recall and reporting bias and may not fully capture symptom variability over time, as participants may have difficulty accurately estimating the frequency, duration, and severity of FOG episodes or sleep disturbances retrospectively. Furthermore, although the PDSS-2 is a validated disease-specific instrument [16], it captures the individual’s subjective perception of sleep disturbances rather than objective sleep metrics such as sleep architecture, sleep efficiency, or specific sleep disorders detectable only through polysomnographic assessment. Similarly, the NFOG-Q measures perceived FOG severity and may not fully correspond to objectively measured gait impairment or actual FOG episodes. We acknowledge that these limitations may have introduced measurement errors and could have attenuated, overestimated, or obscured the true associations between FOG and sleep disturbances, and should therefore be kept in mind when interpreting our findings. Thus, future studies should include objective measures such as wearable technologies or polysomnography alongside patient-reported outcomes to provide more reliable, continuous and accurate data. Sixth, although propensity score matching substantially reduced pre-existing imbalances between groups, the SMD for age in the matched sample slightly exceeded the conventional balance threshold of 0.10 (SMD = 0.140), although the absolute difference was only 1.4 years (68.8 ± 9.6 vs. 70.2 ± 10.5) and the sample would be considered balanced under less stringent thresholds (e.g., SMD ≤ 0.20) commonly adopted in the literature [42]. Nevertheless, this residual imbalance should be considered when interpreting group comparisons. Finally, the matching variable set was deliberately kept minimal to preserve model stability with a limited sample size, to avoid over-adjustment, and to limit the risk of attenuating the FOG-sleep association of interest [42,43]. As a consequence, motor severity and LEDD were not included as matching variables, and residual confounding from these variables cannot be excluded.

## 5. Conclusions

Within the PD+FOG group, FOG severity was positively associated with sleep problems severity, although this exploratory finding warrants confirmation in larger studies. However, no statistically significant differences in sleep problems were detected between PD+FOG and PD−FOG after propensity score matching, although the small sample size may have limited the ability to detect clinically meaningful differences. These findings could be relevant to better understand the relationship between these two disabling and frequent PD manifestations. Finally, PD+FOG participants experienced more severe motor complications and motor symptoms severity compared to PD−FOG participants.

## Figures and Tables

**Table 1:** Demographic and clinical characteristics of the enrolled participants and comparisons between PwPD with and without FOG. *p*-values of Mann–Whitney and Fisher’s exact test are reported, corrected for multiple comparisons using the Benjamini–Hochberg method. For continuous variables, the rank-biserial correlation (r) and its 95% confidence interval (bootstrap, 2000 iterations) are reported as effect size. Significance is marked with an asterisk. EDS: Excessive daytime sleepiness; ESS: Epworth sleepiness scale; FOG: freezing of gait; LEDD: Levodopa equivalent daily dose; MDS-UPDRS: Movement Disorders Society Unified Parkinson’s Disease Rating Scale; mHY: modified Hoehn and Yahr scale; NFOG-Q: New freezing of gait questionnaire; PDSS-2: Parkinson’s disease sleep scale; PwPD: people with Parkinson’s disease.

	PwPD (N = 54)	PD+FOG (N = 27)	PD−FOG (N = 27)	*p*-Value (adj)	r Rank-Biserial	95% CI (Bootstrap)
Age (years)	69.5 ± 10.0	68.8 ± 9.6	70.2 ± 10.5	0.676	0.095	[−0.210, 0.386]
Sex (F)	15 (28%)	8 (30%)	7 (26%)	1.000	-	-
Disease duration (years)	8.7 ± 4.7	8.8 ± 4.5	8.6 ± 5.0	0.676	−0.067	[−0.366, 0.241]
LEDD (mg)	705.9 ± 346.6	822.9 ± 412.3	589.0 ± 215.5	0.091	−0.339	[−0.617, −0.033]
mHY	2.0 (2.0–2.5)	2.0 (2.0–3.0)	2.0 (2.0–2.5)	0.615	−0.111	[−0.379, 0.184]
MDS-UPDRS-I	8.0 (5.0–12.0)	8.0 (5.5–13.5)	7.0 (5.0–12.0)	0.541	−0.151	[−0.460, 0.140]
MDS-UPDRS-II	8.0 (4.0–11.8)	10.0 (7.0–14.0)	6.0 (3.0–9.0)	0.011 *	−0.490	[−0.737, −0.207]
MDS-UPDRS-III	30.0 (24.0–35.0)	32.0 (24.0–37.5)	30.0 (23.5–32.5)	0.533	−0.169	[−0.462, 0.167]
MDS-UPDRS-IV	2.0 (0.0–4.0)	3.5 (1.2–5.0)	0.0 (0.0–2.0)	0.011 *	−0.425	[−0.725, −0.219]
MDS-UPDRS TOT	48.5 (39.2–58.8)	52.0 (43.0–62.5)	44.0 (37.0–51.5)	0.086	−0.361	[−0.632, −0.064]
NFOG-Q	2.0 (0.0–10.0)	10.0 (6.0–13.5)	0.0 (0.0–0.0)	-	-	-
PDSS-2	10.0 (7.0–17.5)	12.0 (7.0–16.0)	10.0 (7.0–19.0)	0.676	−0.077	[−0.381, 0.239]
ESS	6.0 (4.0–9.8)	6.0 (3.5–8.0)	8.0 (4.5–10.5)	0.288	0.240	[−0.070, 0.520]
Fluctuations	31 (57%)	20 (74%)	11 (41%)	0.081	-	-
EDS	14 (26%)	5 (19%)	9 (33%)	0.528	-	-
Sleep disturbances	19 (35%)	9 (33%)	10 (37%)	1.000	-	-

**Table 2:** Correlation between NFOG-Q score and sleep variables. Spearman’s correlation coefficient (ρ) and *p*-values are reported. Significance is marked with an asterisk. ESS: Epworth sleepiness scale; NFOG-Q: New freezing of gait questionnaire; PDSS-2: Parkinson’s disease sleep scale.

	ρ	*p*-Value (adj)
PDSS-2	0.416	0.044 *
ESS	0.190	0.315

## Data Availability

The datasets generated during and/or analyzed during the current study are available from the corresponding authors on reasonable request for privacy reasons.

## Abbreviations

The following abbreviations are used in this manuscript:

PD	Parkinson’s disease
FOG	Freezing of Gait
PwPD	People with PD
PD+FOG	PD with FOG
PD−FOG	PD without FOG
NFOG-Q	New Freezing of Gait Questionnaire
PDSS-2	Parkinson’s Disease Sleep Scale 2
ESS	Epworth Sleepiness Scale
MDS-UPDRS	Movement Disorder Society Unified Parkinson’s Disease Rating Scale
EDS	Excessive Daytime Sleepiness
mHY	modified Hoehn and Yahr scale
LEDD	Levodopa Equivalent Daily Dose
NMS	Non-Motor Symptoms
REM	Rapid Eye Movement
RBD	REM sleep Behavior Disorder
PPN	Pedunculopontine Nucleus
BMI	Body Mass Index
PSQI	Pittsburgh Sleep Quality Index
